# Supplementary material for: Number of teeth and masticatory function are associated with sarcopenia and diabetes mellitus status among community-dwelling older adults: A Shimane CoHRE study
Source: PLoS One. 2021 Jun 2;16(6):e0252625. doi: 10.1371/journal.pone.0252625 (PMC8172058; doi:10.1371/journal.pone.0252625)
Supplement: Attachment — Submitted filename: Response_to_Reviewers0412.docx [file pone.0252625.s001.docx]

**Response to Reviewers**

We thank you for carefully reading our manuscript and for the helpful suggestions. The text revisions per your suggestions have been incorporated and are marked in the manuscript copy.

**Reviewer #1**

***Reviewer’s comment 1.***

Sarcopenia and DM have bidirectional association. However, the authors did not mention it in introduction and discussion.

**Reply:**

Thank you for your important comment. In accordance with your suggestion, we have added this information to the Introduction and Discussion sections of the manuscript.

**<Inserted> Page 4, line 63 (Introduction)**

Recently, numerous bidirectional links between diabetes mellitus and sarcopenia among older adults have been reported. The existence of one condition may increase the risk of developing the other [18]. Therefore, we hypothesized that the maintenance of objective MF and remaining NT as an index of oral health may be related to sarcopenia and diabetes mellitus.

**<Inserted> Page 14, line 207 (Discussion)**

This study has several limitations. First, we used a cross-sectional design, which precludes the possibility of a causal inference among oral health, sarcopenia, or diabetes. Due to the bidirectional relationship between diabetes mellitus and sarcopenia among older adults, the existence of one condition may increase the risk of developing the other [18]. Therefore, our study could not explain the causal relationship among oral health, sarcopenia, and diabetes mellitus.

***Reviewer’s comment 2.***

Because of mutual association between sarcopenia and DM, these variables should be considered as so-founding factors. It should be investigated if number of teeth/masticatory function is associated with sarcopenia independently with DM or not.

**Reply:**

In accordance with your comments 2 and 3, we have revised the methods in the manuscript after we re-analyzed this section. Accordingly, the results and conclusions changed slightly after the correction.

**<Revised> Page 7, line 120 (Methods)**

For the cross-sectional analyses, Poisson regression analyses were performed. This estimated the incidence rate ratio (IRR) and 95% confidence interval (CI) for low levels of handgrip strength or possible sarcopenia with NT (continuous variable) or MF (continuous variable). Logistic regression analyses were performed to estimate the OR and 95% CI for low skeletal muscle mass index, calf circumference with NT (continuous variable), or MF (continuous variable). For all analyses, independent variables were adjusted for sex, age, BMI, smoking, alcohol consumption, and physical activity in Model 1. Model 2 was additionally adjusted for diabetes mellitus. Logistic regression analyses were performed to estimate the OR and 95% CI for the diabetes mellitus outcome with NT (continuous variable) or MF (continuous variable). For all analyses, independent variables were adjusted for sex, age, BMI, smoking, alcohol consumption, and physical activity in Model 1, and additionally adjusted for possible sarcopenia in Model 2.

***Reviewer’s comment 3.***

The number of sarcopenic subjects was too small to be analyzed. The authors segmented 4 or 5 groups according to number of teeth/masticatory function. For example the number of subjects with low handgrip and NT-G3 or MF-Q3 was only 5. I do not think it is too small for this type of statistical analysis.

**Reply:**

In accordance with your comments, we have changed the statistical analysis methods. The number of teeth/masticatory functions was used as the continuous variable as the number of cases in each categorized group was small. We have revised the Methods section. We have also revised the Discussion section based on the modified results.

**<Revised> Page 7, line 120 (Methods)**

For the cross-sectional analyses, Poisson regression analyses were performed. This estimated the incidence rate ratio (IRR) and 95% confidence interval (CI) for low levels of handgrip strength or possible sarcopenia with NT (continuous variable) or MF (continuous variable). Logistic regression analyses were performed to estimate the OR and 95% CI for low skeletal muscle mass index, calf circumference with NT (continuous variable), or MF (continuous variable). For all analyses, independent variables were adjusted for sex, age, BMI, smoking, alcohol consumption, and physical activity in Model 1. Model 2 was additionally adjusted for diabetes mellitus. Logistic regression analyses were performed to estimate the OR and 95% CI for the diabetes mellitus outcome with NT (continuous variable) or MF (continuous variable). For all analyses, independent variables were adjusted for sex, age, BMI, smoking, alcohol consumption, and physical activity in Model 1, and additionally adjusted for possible sarcopenia in Model 2.

**Reviewer #2**

***Reviewer’s comment.***

This is an interesting and significant contribution. The study is well designed and limitations are acknowledged. I recommend having the manuscript revised for language improvement to make it more accurate and engaging. I also recommend you to replace the term elderlies with older adults.

**Reply:**

Thank you for your comments. In accordance with your suggestions, we have replaced the term “elderly” with the term “older adults” throughout the manuscript. In addition, our manuscript has been checked and edited for language by Editage (www.editage.com).
